# Comparative Study of the ORR Activity and Stability of Pt and PtM (M = Ni, Co, Cr, Pd) Supported on Polyaniline/Carbon Nanotubes in a PEM Fuel Cell

**DOI:** 10.3390/nano8050299

**Published:** 2018-05-04

**Authors:** Duanghathai Kaewsai, Mali Hunsom

**Affiliations:** 1 Fuels Research Center, Department of Chemical Technology, Faculty of Science, Chulalongkorn University, 254 Phayathai Road, Bangkok 10330, Thailand; duanghathai.ks@gmail.com; 2 Center of Excellence on Petrochemical and Materials Technology (PETRO-MAT), Chulalongkorn University, 254 Phayathai Road, Bangkok 10330, Thailand; 3 Associate Fellow of Royal Society of Thailand (AFRST), Bangkok 10300, Thailand

**Keywords:** carbon nanotube, polyaniline, PtM catalysts, PEM fuel cell, ORR activity and stability

## Abstract

The oxygen reduction reaction (ORR) activity and stability of platinum (Pt) and PtM (M = Ni, Co, Cr, Pd) supported on polyaniline/carbon nanotube (PtM/PANI-CNT) were explored and compared with the commercial Pt/C catalyst (ETEK). The Pt/PANI-CNT catalyst exhibited higher ORR activity and stability than the commercial Pt/C catalyst even though it had larger crystallite/particle sizes, lower catalyst dispersion and lower electrochemical surface area (ESA), probably because of its high electrical conductivity. The addition of second metal (M) enhanced the ORR activity and stability of the Pt/PANI-CNT catalyst, because the added M induced the formation of a PtM alloy and shifted the *d*-band center to downfield, leading to a weak chemical interaction between oxygenated species and the catalyst surface and, therefore, affected positively the catalytic activity. Among all the tested M, the addition of Cr was optimal. Although it improved the ORR activity of the Pt/PANI-CNT catalyst slightly less than that of Pd (around 4.98%) in low temperature (60 °C)/pressure (1 atm abs), it reduced the ESA loss by around 14.8% after 1000 cycles of repetitive cyclic voltammetry (CV). In addition, it is cheaper than Pd metal. Thus, Cr was recommended as the second metal to alloy with Pt on the PANI-CNT support.

## 1. Introduction

Among the several types of fuel cells, proton exchange membrane (PEM) fuel cells are one of the most attractive types because they possess several advantages, including a high efficiency, clean operation and rapid start-up and shut-down. However, the PEM fuel cells commercialization for actual application is limited by their high cost and low stability of the electrodes [1]. Generally, Pt on carbon support (Pt/C) is most widely used as the catalyst for the anode and cathode in PEM fuel cells. Among various types of support materials, carbon black (Vulcan XC-72) has been used widely in PEM fuel cells because of its high electrical conductivity and high specific surface area [2,3], but it suffers from electrooxidation under fuel cell operating conditions resulting in the loss of catalytic activity after long-term operation [4,5]. Thus, the carbon nanotubes (CNTs) are a seemingly ideal candidate material for a catalyst support because of their unique properties, such as a long aspect ratio for efficient electron transfer, high surface area, good electrical conductivity and high chemical stability [6,7]. However, the surface of CNTs is inert and hydrophobic, giving a poor compatibility with metal nano-particles (NPs). Thus, their surface is usually modified prior to use to improve the surface chemistry/property for immobilizing the metal catalyst NPs. The most common surface modification method to create functional groups on the surface of the CNTs is the acid treatment [8,9,10]. However, this treatment sometimes decreased electrical conductivity of the CNTs structure [9,11,12].

To alleviate this problem, various types of conductive polymer were wrapped on the CNTs surface. For example, wrapping of the CNTs by polystyrene sulfonate provided a good dispersion of Pt NPs on its surface [13]. The poly(benzimidazole) (PBI)-wrapped CNTs provided a homogeneous Pt immobilization onto the CNT surface because of the coordination of the Pt ions with the PBI molecule resulting in a high catalyst utilization [14]. During the synthesis of CNTs wrapped by pyridine-containing PBI (PyPBI), the PyPBI wrapping acted as a glue for immobilizing the Pt NPs, and so resulted in a highly homogeneous dispersion of Pt NPs on the CNT surface. Thus, the Pt NPs anchored on the PyPBI-wrapped CNTs provided a high electrochemically active surface area (ESA) and a better fuel cell performance [15,16]. The wrapping of CNTs with polypyrrole (PPy) prevented the agglomeration of Pt NPs onto the surface of CNTs and resulted in an improvement in the fuel cell performance [17]. In particular, the wrapping of CNTs with polyaniline (PANI), which can serve as a dispersant and stabilizer for immobilizing of Pt NPs, led to enhanced surface properties, such as the electrical conductivity, chemical resistance and surface area [18,19,20]. Moreover, it was reported that the PANI-decorated carbon material supported Pt help to improve the electrode stability in fuel cells [21,22].

As mentioned previously, the supported Pt NPs have been widely used as a catalyst in PEM fuel cells. However, Pt is expensive and rare, thus various PtM alloys have been intensively studied, such as PtCr, PtFe, PtCo, PtNi, PtCu, PtMo, and PtPd [23,24,25], to enhance the ORR kinetics and stability in the electrodes. The addition of a second metal into Pt to form PtM alloy catalyst improved the electronic and geometric parameters of the Pt metal (i.e., metal particle size, surface structure, and shortening of Pt-Pt bond distance) [26]. As reported in our previous work, the addition of an appropriate PANI content (10 wt %) can enhance the hydrophilic property of CNTs, and consequently positively affected the ORR activity of the PtCo/PANI-CNT catalyst [27]. In this work, 10 wt % of PANI was wrapped on the CNT surfaces and was then used as the support for Pt and PtM catalysts (M = Ni, Co, Cr, Pd). The ORR activity and stability were studied in both an acid solution and PEM fuel cell at room temperature and ambient pressure in direct comparison with the commercial Pt/C catalyst (ETEK) for reference. The novelty of this work is the comparative study of the ORR activity and stability of several Pt alloy catalysts supported on the polyaniline-wrapped carbon nanotube, which had never been studied before.

## 2. Experimental

### 2.1. Preparation of the PANI-CNT Support

In this study, multi-walled CNTs purchased from Nano Generation Co. Ltd. (Chiang Mai, Thailand) with diameters of 20–40 nm were used as the catalyst support. Prior to use, their surface was treated in a 7:3 (*v*/*v*) ratio of 12 M mixed HNO_3_ (QRëC) and H_2_SO_4_ (QRëC) by dispersing 0.1 g of the CNTs in mixed acid and stirring at 250 rpm for 6 h. The carbon slurry was held for 18 h and separated afterward from the acid solution by filtration and rinsed many times by deionized (DI) water until the pH of filtrate was constant (pH ~5). The ready-to-use CNTs were obtained after drying at 110 °C for 2 h [28].

To prepare the polyaniline (PANI)-wrapped CNT (PANI-CNT), the aniline monomer (99.99%, Panreac, Lyon, France) was used as the PANI precursor. It was distilled at 130 °C to remove the inhibitor [29]. The PANI content wrapped on the CNT surfaces was fixed at 10 wt % as previously reported [27], and was achieved by dispersing 1.0 g of treated CNT in 100 mL of 2 M HCl (37 wt.%, QRëC, Selangor, Malaysia) solution and sonicated at room temperature for 2 h. Then, 0.1 mL of aniline monomer was added slowly under sonication at 30 °C. To activate the polymerization of aniline monomer to PANI, approximately 20 mL of 0.2 M ammonium persulfate ((NH_4_)_2_S_2_O_8_, Univar, Ajax Finechem, Auckland, New Zealand) were slowly added drop wise into the mixture with constant stirring rate in an ice bath at 0–5 °C for 3 h. Subsequently, the dark suspension was filtered and washed several times with DI water until the filtrate became colorless. The ready-to-use PANI-CNT support with 10 wt % PANI was obtained after drying in an oven at 80 °C for 24 h.

### 2.2. Synthesis of PtM/PANI-CNT Catalysts

The Pt/PANI-CNT and PtM/PANI-CNT catalysts, where M is the second metal (Ni, Co, Cr and Pd), were synthesized by the impregnation-seeding technique [30]. For the preparation of PtNi/PAN-CNT catalyst, 0.0498 g of H_2_PtCl_6_ 6H_2_O (Alfa Aesar, Massachusetts, USA) and 0.0253 g of NiCl_2_ 6H_2_O (QRëC, Selangor, Malaysia) were dispersed together in DI water to form the mixed-metal solution. At the same time, 0.1 g of PANI-CNT was dispersed in DI water, sonicated at 70 °C for 0.5 h and then adjusted to pH 2 using 6 M HCl (37 wt. %, QRëC, Selangor, Malaysia) to obtain the carbon slurry. Consequently, 10 vol % of the PtNi solution was mixed thoroughly with the carbon slurry, sonicated at 70 °C for 0.5 h and then reduced slowly by the addition of 20 mL of 0.2 M NaBH_4_ (Loba Chemie, Mumbai, India). The solid catalyst was harvested by filtration and then rinsed with DI water. The obtained wet catalyst was further used as the seed catalyst for the remaining catalyst by dispersing in 5 mL DI water, sonicated for 0.5 h and then added into the remaining 90% (*v*/*v*) PtNi solution. Subsequently, the mixture was reduced again by the addition of 20 mL of 0.2 M NaBH_4_ under sonication to obtain the catalyst slurry. The ready-to-use PtNi/PANI-CNT catalyst was then obtained after filtration, washed with DI water until neutral in pH, and finally dried overnight at 110 °C. For the other PtM/PANI-CNT catalysts, a similar preparation procedure was performed only using 0.0252 g of CoCl_2_ 6H_2_O (Univar, Ajax Finechem, Auckland, New Zealand), 0.0424 of g Cr(NO_3_)_3_ 9H_2_O (Himedia) or 0.0188 g of PdCl_2_ (Fluka) as the Co, Cr or Pd precursors for the PtCo/PANI-CNT, PtCr/PANI-CNT and PtPd/PANI-CNT catalysts, respectively. According to this procedure, a Pt:M atomic ratio of 3:1 will be obtained.

### 2.3. Preparation of the Carbon Sublayer and Membrane Electrode Assembly (MEA)

The carbon sublayer was first prepared by mixing 0.5 mL of DI water with 1.334 μL of 60% (*w*/*w*) polytetrafluoroethylene (Aldrich , St. Louis, MO, USA ) under sonication at room temperature for 0.5 h, followed by the addition of 1.0 mL of *i*-propanol (C_3_H_8_O, QRëC, Selangor, Malaysia) and sonicated for 0.5 h. Then, 0.018 g of treated carbon powder (Vulcan XC-72) was added and sonicated for 0.5 h to get the carbon ink. The obtained ink was brushed onto a 2.25 × 2.25 cm^2^ gas diffusion layer (GDL, ETEK) and subsequently dried at 80 °C for 2 min to remove the excess solvent. The coating of sublayer ink was repeated to obtain a sublayer loading of 2.0 mg/cm^2^. Finally, the ready-to-use sublayer ink-coated GDL was dried at 300 °C for 1 h.

The catalyst-coated membrane was then prepared by dispersing 25 mg of the prepared catalyst in 1 mL DI water under sonication for 1 h. Then, 1.0 mL of *i*-propanol was slowly added and sonicated again for 1 h, then 0.44 mL of Nafion solution (5 wt %, Fluka, Buchs, Switzerland) *was* added and sonicated for 2 h at 30 °C to obtain the catalyst ink. The ink was then sprayed on the surface of the 5-cm^2^ treated membrane (Nafion 115, Electrochem, Inc., Woburn, MA, USA) using a spray gun (Crescendo, Model 175-7TM). The similar procedure was done for the second surface of treated membrane, while the commercial Pt/C (ETEK) was used instead of the PtM/PANI-CNT catalyst. The catalyst loading on each membrane surface was fixed at 0.15 mg/cm^2^.

**Figure 1:**
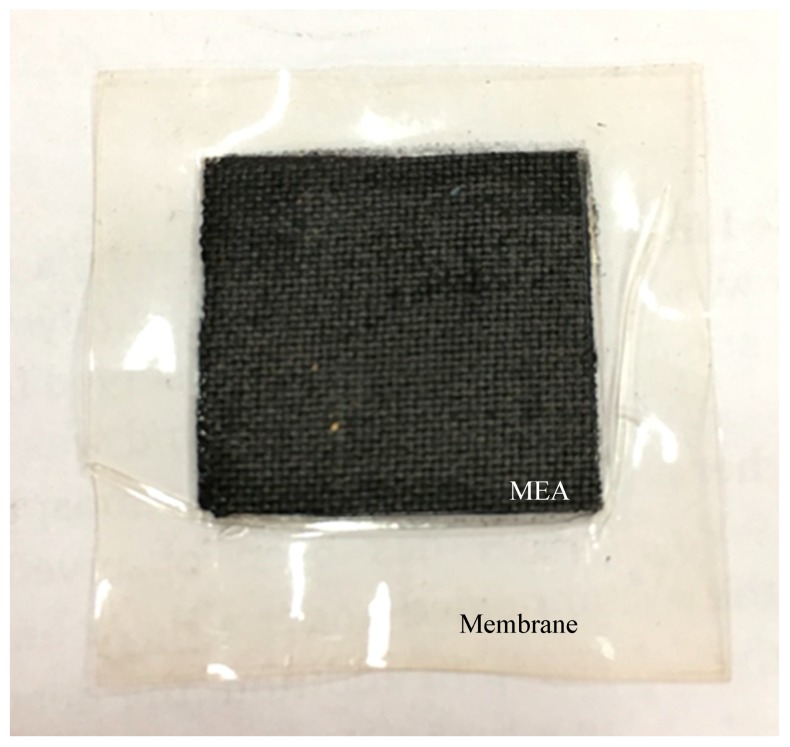
Example of the obtained MEA.

Finally, to get the membrane assembly (MEA), two sheets of sublayer ink-coated GDL were put on both sides of catalyst-coated membrane and then pressed by a compression mold (LP20, Labtech, Tampa, FL, USA) at 137 °C with a pressure of 65 kg_f_/cm^2^ for 2.5 min. The external feature of the obtained MEA is exhibited in Figure 1.

### 2.4. Catalyst and Electrode Characterization

The diffraction peaks of the all prepared catalysts was observed by X-Ray diffraction (XRD) on a D8-Discover-Bruker AXS machine equipped with Cu Kα. The average particle size and distribution were estimated by averaging not less than 100 particles randomly distributed in transmission electron microscopy (TEM) images observed on a JEOL JEM-2100. The loading content of the respective metal on the PANI-CNT support was examined by field emission scanning electron microscopy (SEM) and energy dispersive X-ray spectrometry (EDX) on a JEOL JSM-7610F. The chemical state and the surface composition of all prepared catalysts were monitored by X-ray photoelectron spectroscopy (XPS) with PHI 5000 VersaProbeII equipment and an Al Kα monochromatic X-ray source (*hν* = 1486.6 eV).

The ESA of all prepared catalysts was estimated by the H_2_ stripping method in N_2_-saturated 0.5 M H_2_SO_4_ solution. Initially, the catalyst layer was prepared by dripping 1.0 μL of the respective catalyst onto the glassy carbon electrode (GCE) of 2 mm diameter, assembled with the rotating disc electrode (RDE) and connected with the Potentiostat/Galvanostat (Autolab, PG Stato 30) as the working electrode, while a saturated calomel electrode (SCE) and Pt rod were employed as the reference and counter electrodes, respectively. The CV was performed at room temperature in the potential range of −0.2 V to +0.8 V at a scan rate of 20 mV/s. The ESA of each catalyst was calculated from the H_2_ desorption peak according to Equation (1) [31]:

(1) $$\mathrm{ESA}=\frac{Q_{H}}{[Pt]q_{H}}$$

The in-plane conductivity of all catalysts was measured using a four-point probe instrument (Jandel, RM3-AR) at room temperature and pressure (65% humidity), which can be calculated by Equation (2):

(2) $$\sigma =\frac{I}{2\pi sVt}$$

### 2.5. ORR Activity and Stability Tests

The ORR activity of all catalysts was tested by linear sweep voltammetry (LSV) with the RDE in an acid solution (0.5 M H_2_SO_4_) and in a PEM fuel cell under H_2_/O_2_ environment. For the first test, 1.0 μL of each respective catalyst ink was dripped onto a GCE of 2 mm diameter, connected with the RDE and put in 0.5 M O_2_-saturated H_2_SO_4_. It was then combined with the Potentiostat/Galvanostat (Autolab, PG Stato 30) as the working electrode. A Pt rod was used as the counter, while a SCE was employed as reference electrodes. The O_2_ (99.99%, Praxair, Danbury, CT, USA) was supplied to the system during the test to ensure the saturation of O_2_ in the H_2_SO_4_ solution. The test was performed with LSV using a potential in the range of +0.10 V to +0.80 V at different rotation rates (500–2000 rpm) and a scan rate of 20 mV/s, controlled by the Nova 1.1 software package from Metrohm. For the second test, the MEA was assembled on a commercial single cell hardware (Electrochem, Inc., Woburn, MA, USA) and tested in an in-house test station. The commercial Pt/C catalyst (ETEK) was used as the anode, while the prepared PtM/PANI-CNT catalyst was used as the cathode. The H_2_ (99.99%, Praxiar) and O_2_ (99.9999%, Praxair, Danbury, CT, USA) were supplied at the same flow rates of 100 sccm to the fuel cell, which was operated at 60 °C under atmospheric pressure. Prior to recording the current density-potential data, the fuel cell was conditioned by operating at a current density greater than 700 mA/cm^2^ for at least 6–12 h. 

The stability of the catalysts was monitored from the change in the ESA of the H_2_ desorption peak from the H_2_ stripping method in N_2_-saturated 0.5 M H_2_SO_4_ after applying the repetitive CV during the potential of −0.2 to +0.8 V, similar to the procedure explained in Section 2.4.

## 3. Results and Discussion

### 3.1. Property of Pt/PANI-CNT and PtM/PANI-CNT Catalysts

**Figure 2:**
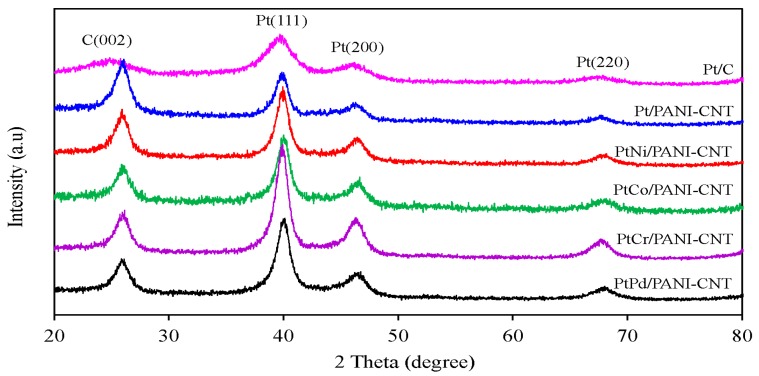
Representative of XRD patterns of the different catalysts.

Figure 2 shows representative XRD patterns of the commercial Pt/C (ETEK) and the synthesized Pt/PANI-CNT and PtM/PANI-CNT catalysts. It can be seen that the commercial Pt/C catalyst exhibited a graphitic structure of carbon support at 2θ of 24.85° and also exhibited the characteristic diffraction peaks of the face-centered cubic (fcc) phase of Pt at 2θ of 39.69°, 46.21° and 67.85°, corresponding to the Pt(111), Pt(200), and Pt(220) planes, respectively. The XRD pattern of the Pt/PANI-CNT catalyst also exhibited the three main characteristic fcc peaks of metallic Pt at 2θ of 39.85°, 46.30° and 67.73°, together with the diffraction peak of the CNTs structure at 2θ of 26.08°. Four XRD peaks, including a diffraction peak of the CNTs support and the three diffraction peaks of Pt were also observed for each respective PtM/PANI-CNT catalyst. The lattice parameters of PtM/PANI-CNT catalysts were found to be lower than the Pt/PANI-CNT and Pt/C catalysts (Table 1), indicating the formation of the PtM alloy on the PANI-CNT support. This is because the added M can intercalate into the fcc structure of the Pt particles, resulting in a reduction in the Pt-Pt atomic distance, which consequently allowed a fast adsorption/desorption of oxygen molecules to proceed the reaction with an improved ORR activity [32].

**Figure 3:**
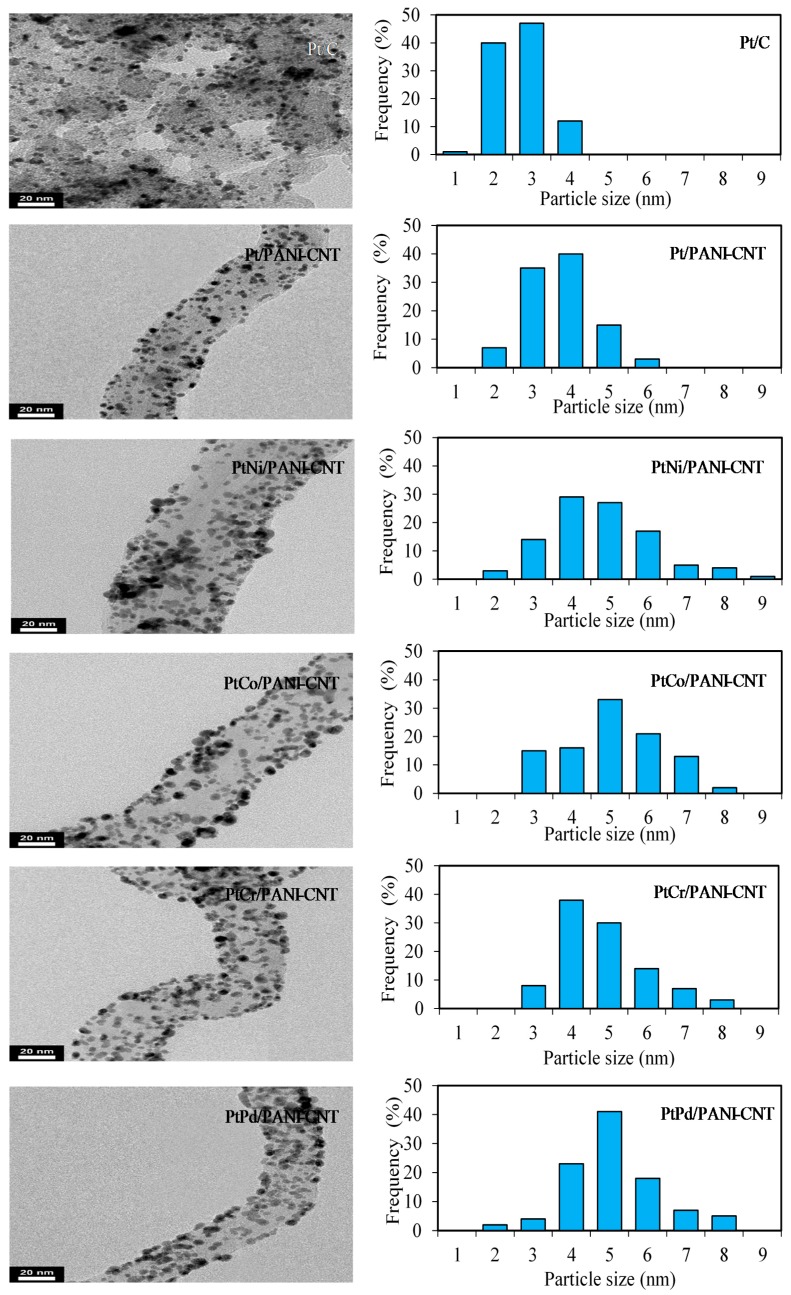
(**Left**) Representative of TEM images of the different catalysts; and (**Right**) their respective histograms of the anhydrous metal particle size distribution.

The average crystallite size of all the prepared catalysts was estimated from Scherrer’s equation [33]. The crystallite size of Pt in the Pt/PANI-CNT catalyst was larger than that in the Pt/C catalyst (Table 1), consistent with the particle sizes observed in the TEM analysis (Figure 3). This suggested a slight agglomeration of Pt particles on the PANI-CNT support, which was probably because of the different quantity of oxygen containing surface groups on surface of PANI-CNT in comparison to that on the carbon. A high density of oxygen containing surface groups can enhance an increased catalyst crystallite and/or particle size by diminishing the number of surface basic sites, which are the centers for the strong adsorption of PtCl_6_^2−^ [34], decreasing the metal-support interaction [35] and hindering the Pt precursor reduction [36]. The particle sizes of all PtM on the PANI-CNT support were nearly the same (Table 1), suggesting that alloying of Pt with different M did not induce an agglomeration of catalyst particles on the PANI-CNT surface.

**Figure 4:**
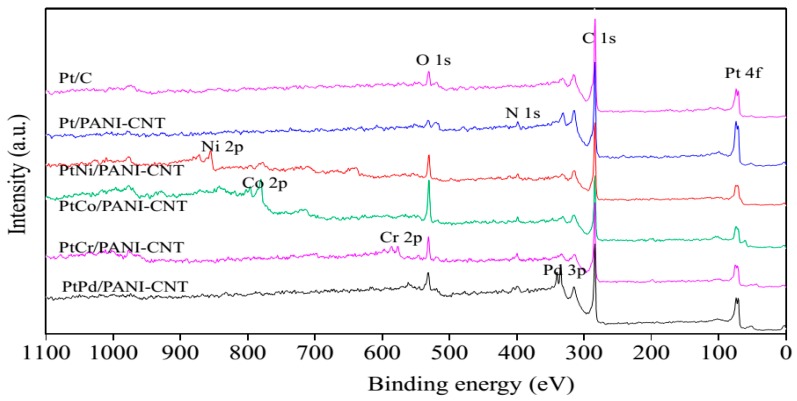
Representative of XPS spectra of the different catalysts.

From the XRD patterns, the characteristic peaks of each respective M did not appear, probably due to their presence in small quantities or in amorphous forms. Thus, XPS and SEM-EDX analyses were employed to detect the presence of these metal elements. As demonstrated in Figure 4, besides the presence of the basic elements, including C 1s (from support), O 1s (from the surface functional groups of the support), N 1s (from the amine group of PANI) and Pt 4f, in all samples in the survey XPS spectra, the signals of Ni 2p, Co 2p, Cr 2p and Pd 3d were, respectively, observed in the PtNi/PANI-CNT, PtCo/PANI-CNT, PtCr/PANI-CNT and PtPd/PANI-CNT catalysts. This evidenced the presence of the respective M in the PtM/PANI-CNT catalysts.

**Figure 5:**
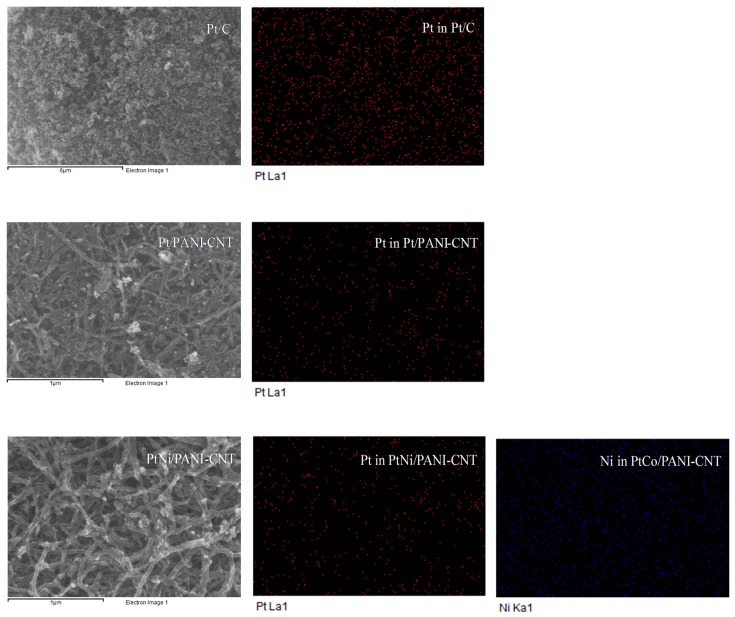

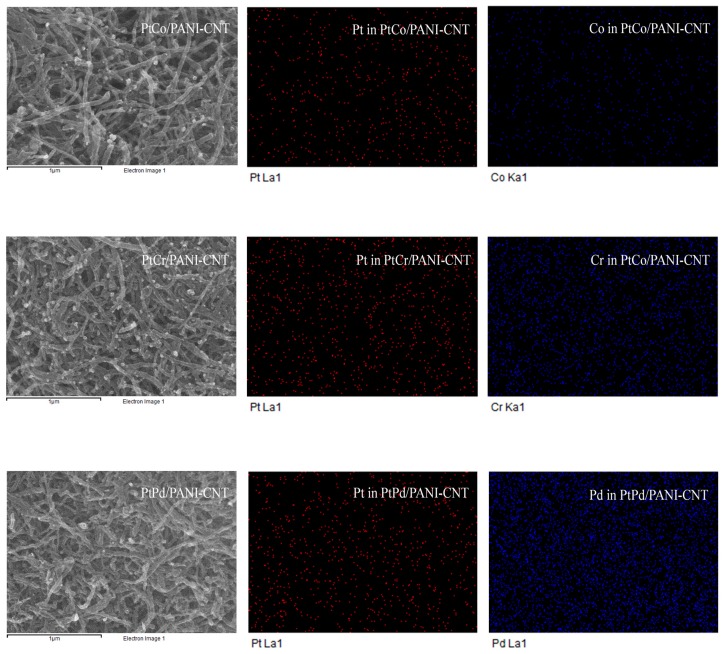
(**Left**) Representative SEM; and (**Middle**,**Right**) EDX mapping pattern of the catalysts.

The appearance of the dispersed Pt and M metals along the support surface was clearly observed in the respective SEM-EDX analysis, as exhibited in Figure 5, in which both Pt and M were uniformly distributed on the support surface. This was due to the presence of the N-group on the PANI surface that could bond directly with metal ions [27]. The quantitative metal dispersion along the support was determined by polynomial equations [37], as shown in Equations (3)–(5):

(3) $$N_{T}=\frac{2\pi }{3}{\left(\frac{D}{a}\right)}^{3}$$

(4) $$N_{T}=\left(\frac{10}{3}\right)l^{3}-5l^{2}+\left(\frac{11}{3}\right)l-1$$

(5) $$N_{S}=10l^{2}-20l+12$$

As summarized in Table 1, the highest catalyst dispersion (32.76%) was observed in the commercial Pt/C catalyst. A decreased level of Pt dispersion was observed when PANI-CNT was used as the support. The dispersions of PtM on the PANI-CNT support fluctuated slightly within the range of 20.86–21.73%. The atomic ratios of Pt:M were around 3:1, in agreement with the set value during the preparation stage. In addition, from the SEM-EDX analysis, the metal loadings in the prepared catalysts were nearly the same, in the range of 15.2–17.8% (Table 1).

**Figure 6:**
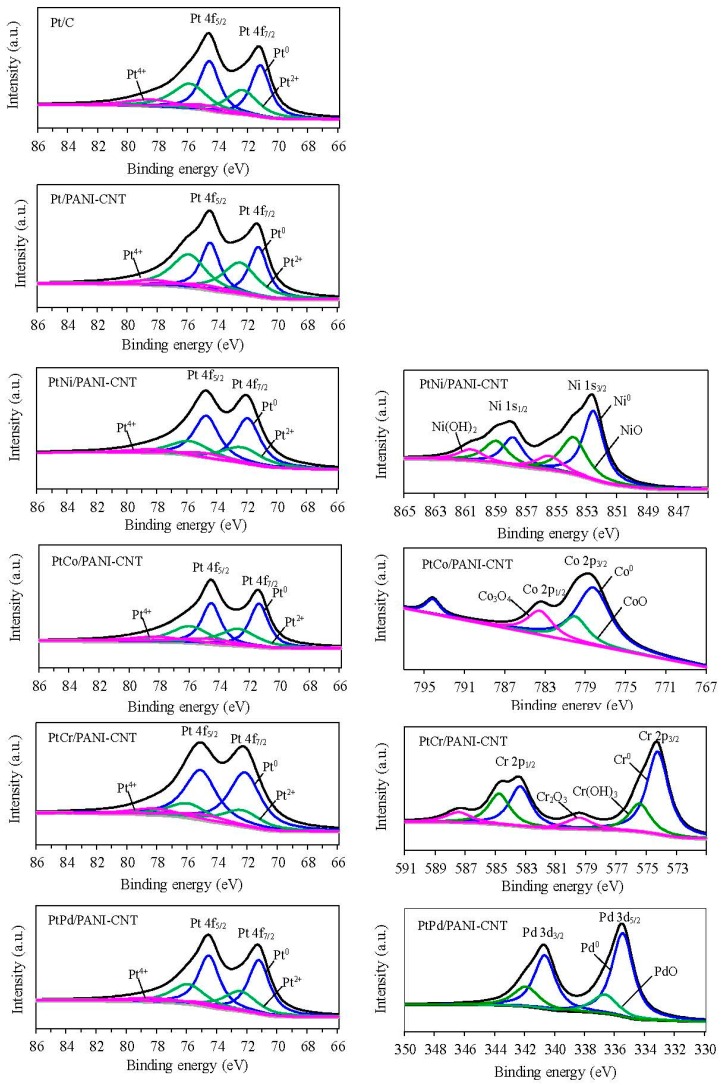
Representative of XPS spectrum of the: (**Left**) Pt 4f; and (**Right**) Ni, Co, Cr or Pd signals for the different catalysts.

Figure 6 presents the Pt 4f core level spectra of the prepared catalysts. After deconvolution with XPSpeak 41 software using a symmetrical Guass distribution, the two main characteristic peaks observed at a binding energy of around 71–73 eV and 74–77 eV were the spin orbital splitting of Pt4f_7/2_ and Pt4f_5/2_, respectively [38,39]. The binding energies of Pt4f for Pt/C, Pt/PANI-CNT, PtNi/PANI-CNT, PtCo/PANI-CNT, PtCr/PANI-CNT and Pt/PANI-CNT were 71.25, 71.38, 72.50, 71.38, 72.75 and 71.50, respectively, for Pt 4f_7/2_ and 74.63, 74.38, 74.50, 74.50, 75.88 and 74.75, respectively, for Pt 4f_5/2_. It can be seen that the binding energy of all PtM/PANI-CNT catalysts exhibited a blue shift relative to the Pt/PANI-CNT catalyst, confirming the formation of the PtM bimetallic alloy NPs [40]. In addition, it is reported that the binding energy is strongly correlated with the adsorption/desorption capability of reactive species on the catalyst surface [41]. The positively shifted binding energy of Pt indicated a downshift in the *d*-band center [42], which would lead to a weak chemical interaction between oxygenated species and catalyst surface and, therefore, positively affect the catalytic activity [43]. The different Pt electronic structures among the PtM/PANI-CNT catalysts mainly originated from the different electronegativity of M to donate an electron to Pt [43].

The deconvolution of both main peaks of the prepared catalysts showed the prevalence of Pt in metallic and oxidized forms. As summarized in Table 2, both Pt/PANI-CNT and PtM/PANI-CNT catalyst exhibited high Pt content in comparison with the Pt/C. This is because the N-functional groups on CNT surface could facilitate the formation of Pt^0^ [44]. Among all prepared PtM/PANI-CNT catalysts, the prevalence of Pt^0^ was slightly deviation in a narrow range of 56.82–60.12%. For M, they also occurred in metallic, oxide and sometimes hydroxide forms, and the prevalence of M^0^ was ranked in the order of Pd (74.86%) > Cr (59.37%) > Co (54.67%) > Ni (48.37%) in the PtM/PANI-CNT catalysts.

### 3.2. Electrochemical Properties of Pt/PANI-CNT and PtM/PANI-CNT Electrodes

As summarized in Table 1, the in-plane electrical conductivities of the Pt/PANI-CNT and PtM/PANI-CNT electrodes in air at 65% humidity were nearly the same, which were higher than the commercial Pt/C catalyst. This suggested that the PANI-CNT support exhibited a higher electrical conductivity than the commercial carbon support and the formation of the respective PtM alloys did not significantly affect the electrical conductivity of PtM/PANI-CNT electrode. With respect to the ESA of all the prepared catalysts, the ESA of Pt/PANI-CNT was slightly lower than that of the commercial Pt/C catalyst (Table 1), probably due to its large particle size. Although the PtM/PANI-CNT catalysts had almost similar particle sizes, they exhibited a lower ESA than the Pt/PANI-CNT catalyst. This might be due to the presence of a lower quantity of Pt in all the PtM/PANI-CNT catalysts (~71.3 to 74.63% Pt) compared to in the Pt/PANI-CNT catalyst (100% Pt). For the PtM/PANI-CNT catalysts, their ESA was ranked in the order of PtPd/PANI-CNT > PtCr/PANI-CNT > PtCo/PANI-CNT > PtNi/PANI-CNT, which probably reflects the effect of the quantitative existence of the active single plane of Pt. That is, due to the nature of the method used to estimate the ESA (H_2_ adsorption method), the atomic hydrogen will adsorb only on the single crystal planes of Pt (100, 110 or 111) [45]. However, from the XRD analysis, only the Pt(111) plane was found in the prepared samples, suggesting that the addition of Pd facilitated the formation of a single crystal plane of Pt (111) on the PANI-CNT more than the addition of Ni, Co or Cr.

### 3.3. ORR Activity Test

#### 3.3.1. ORR Activity in Acid Solution

**Figure 7:**
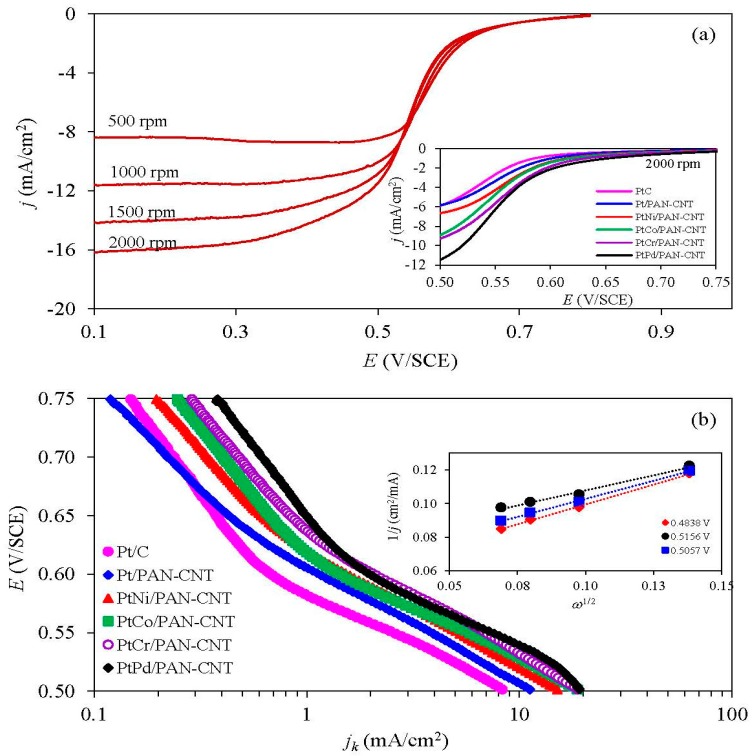
Representative of: (**a**) LSV with steady-state polarization curves for ORR at 2000 rpm (inset figure); and (**b**) kinetic current density with example of Levich plot (inset figure) of the different catalysts.

The ORR activity of all the catalysts was initially investigated using a RDE in O_2_-saturated 0.5 M H_2_SO_4_ at different rotation rates of 500 rpm to 2000 rpm. A well-defined current density-potential curve was observed (Figure 7a), including: (*i*) a kinetics-controlled region (+0.80 V to +0.62 V); (*ii*) an intermediate region of mixed control (+0.62 V to +0.52 V); and (*iii*) a diffusion-controlled region (+0.52 V to +0.20 V). At the same rotation rate, the onset potential of the Pt/PANI-CNT and all PtM/PANI-CNT catalysts were shifted to a higher potential compared to the commercial Pt/C catalyst (inset of Figure 7a), and they could be ranked in the order of PtPd/PANI-CNT > PtCr/PANI-CNT > PtCo/PANI-CNT > PtNi/PANI-CNT > Pt/PANI-CNT, presumably indicating a direct trend in the ORR activity.

To determine the exact ORR activity of each catalyst, the obtained current density–potential data of each catalyst was plotted against the rotation rates using the Koutecky-Levich equation [46], as shown in Equation (6):

(6) $$\frac{1}{j}=\frac{1}{j_{k}}+\frac{1}{B\omega^{1/2}}$$

A plot of 1/*j* vs. 1/ω provided a straight line with a slope of 1/*B* and an intercept with 1/*j_k_* (Inset of Figure 7b). The value of *B* can be used to determine the number of electrons transferred in the ORR according to Equation (7), while the value of *j_k_* indicated the ORR activity of catalysts.

(7) $$B=0.62n_{e}FD^{2/3}\nu^{-1/6}C$$

As shown in Figure 7b, the *j_k_* altered as the change of applied potential. Over the whole investigated potential range, the trends of *j_k_* for the different catalysts were ranked in the order of PtPd/PANI-CNT > PtCr/PANI-CNT > PtCo/PANI-CNT > PtNi/PANI-CNT > Pt/PANI-CNT > Pt/C, suggesting that the PtPd/PANI-CNT catalyst was more active than the other catalysts. This result is consistent with the ORR activity presumed by the positive potential shift of the LSV curve. In addition, from this calculation, the number of transferred electrons varied from 3.33 to 4.06, indicating that the ORR reaction occurred via (or nearly via) a four-electron pathway.

#### 3.3.2. ORR Activity in the PEM Fuel Cell

**Figure 8:**
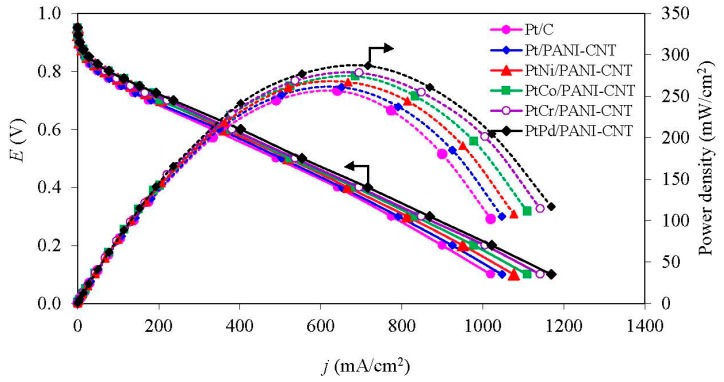
Representative of ORR activity of the Pt- and supported PtM catalysts in a single PEM fuel cell at 60 °C under a H_2_/O_2_ environment and ambient pressure.

The activity of all the PtM/PANI-CNT catalysts was also evaluated in a PEM fuel cell under a H_2_/O_2_ atmosphere at low temperature of 60 °C and ambient pressure. The open circuit voltage (OCV) of all the prepared catalysts was similar (Figure 8), ranging 0.949–0.963 V. This suggested that the different PtM alloy catalysts did not markedly affect the OCV of the single cell. During the activation-controlled region (<100 mA/cm^2^), all PtM/PANI-CNT catalysts provided a higher current density at 0.9 V (*j*_0.9V_) than the Pt/PANI-CNT and commercial Pt/C catalyst (Table 3). This is probably because the presence of the M in the PtM alloy catalyst modifies the geometric (Pt-Pt interatomic distance) and electronic (*d*-band vacancy) structure of the Pt catalyst. The *j*_0.9V_ of all PtM/PANI-CNT catalysts was ranked in the order of PtPd/PANI-CNT > PtCr/PANI-CNT > PtCo/PANI-CNT > PtNi/PANI-CNT, consistent with the ESA trends (Table 2) and the amount of Pt^0^ observed by XPS analysis (Table 2), resulting in a high oxygen adsorption on the catalyst surface [47]. 

At the medium-to-high current density (the ohmic-controlled region), a similar trend of catalytic activity was still observed. That is, the Pt/PANI-CNT catalyst provided a higher current density than the commercial Pt/C catalyst, but lower than the PtM/PANI-CNT catalysts at all potentials. The PtPd/PANI-CNT catalyst exhibited the highest current density (402 mA/cm^2^) and power density (241 mW/cm^2^) at 0.6 V. The kinetic and mass transport parameters of all catalysts in the PEM fuel cell were then estimated from a nonlinear least squares (NLLS) method [48], as expressed in Equation (8):

(8) $$E\;=\;E_{0}\;-\;b\log j\;-\;jR\;-\;m\;\exp\;\left(nj\right)$$

where $E_{0}\;=\;E_{r}\;+\;b\;\log j_{0}$.

From this method, the coefficient of determination (*R*^2^) for the prepared catalysts was found to be greater than 0.9995, indicating that all the fitted models could adequate to predict the experimental data. The intrinsic Tafel slopes (*b*) of the catalysts towards the ORR fluctuated in a narrow range between −56.0 and −57.7 mV/dec under identical testing conditions, which was attributed to the variation in the interphase conditions in the presence of the different PtM alloy catalysts. The PtPd/PANI-CNT catalyst provided the maximum exchange current density for the ORR (*j*_0_), being some 2.23-, 2.08-, 1.92-, 1.94- and 1.17-fold higher than that for the Pt/C, Pt/PANI-CNT, PtNi/PANI-CNT, PtCo/PANI-CNT and PtCr/PANI-CNT catalysts, respectively. The total resistance of all PtM/PANI-CNT catalysts fluctuated slightly in the range of 0.5150–0.5200 Ω cm^2^, which were lower than those of the Pt/C and Pt/PANI-CNT catalysts. The mass-transport overpotentials (monitored in terms of parameter *n*) of the catalysts were ranked in the order of Pt/C > Pt/PANI-CNT > PtNi/PANI-CNT > PtCo/PANI-CNT > PtCo/PANI-CNT > PtPd/PANI-CNT. This indicated that the PtPd/PANI-CNT catalyst required the lowest potential to drive the reaction. In addition, the PtPd/PANI-CNT catalyst had the highest mass transport limitation, as observed from the highest value of parameter *m* compared to the other five catalysts.

### 3.4. Stability Test

**Figure 9:**
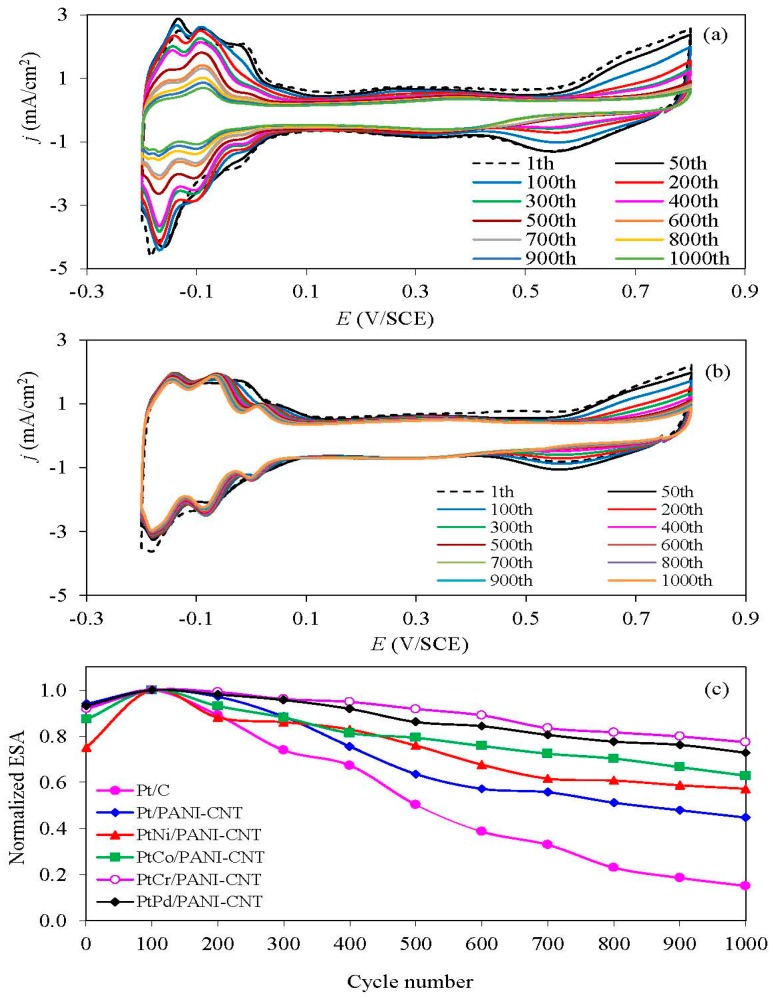
Representative of repetitive CV of: (**a**) Pt/C and (**b**) PtPd/PANI-CNT; and (**c**) the normalized ESA of the supported catalysts in N_2_-saturated 0.5 M H_2_SO_4_ at a scan rate of 20 mV/s.

The stability of the prepared Pt/PANI-CNT and PtM/PANI-CNT catalysts and the commercial Pt/C one was evaluated using repetitive CV in N_2_-saturated 0.5 M H_2_SO_4_ over a potential range of −0.2 and +0.8 V/SCE at a scan rate of 20 mV/s. A representative repetitive CV trace is shown in Figure 9a,b, where a well-defined hydrogen adsorption/desorption peak was observed. The quantitative ESA of the catalysts was then determined via Equation (1) at a particular CV cycle. In terms of the normalized ESA, the Pt/PANI-CNT catalyst exhibited a lower ESA loss than the commercial Pt/C catalyst, suggesting its higher stability. This is because the conductive PANI can help to reconfigure the catalyst-support interaction [27] and sometimes partially hinders the exposure of the support with the generated H^+^, resulting in a low corrosion of the catalyst support. All PtM/PANI-CNT catalysts exhibited a smaller ESA loss than the Pt/PANI-CNT catalyst, probably due to a low level of Pt aggregation during alloy formation. Among all the PtM/PANI-CNT catalysts, the addition of Cr enhanced the Pt catalyst stability more than the addition of Ni, Co or Pd, consistent with the previously defined activity/stability level of the PtM alloys [49], in which Cr was classified as a stable metal once composited with Pt to form the PtCr alloy. This likely reflects that PtCr had an appropriate metallic-ordered state to resist the acid environment.

**Figure 10:**
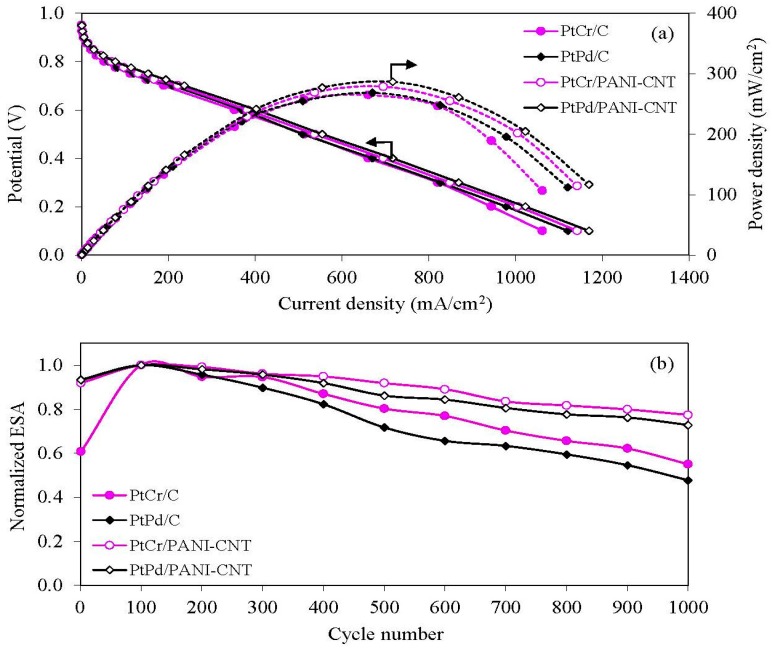
Representative of comparative: (**a**) performance in PEM fuel cell; and (**b**) stability in 0.5 M H_2_SO_4_ of the different catalysts.

To confirm the activity and stability of the support materials, two types of Pt alloy (PtCr and PtPd) supported on the PANI-CNT and commercial carbon black (Vulcan XC-72) were tested comparatively. As shown in Figure 10, the PANI-CNT support provided a higher ORR activity and stability than the commercial carbon support, which could be attributed to the unique properties of multiwall CNTs in comparison with a carbon support, including an excellent electrical conductivity, high surface area, high chemical stability and high amount of mesopores, which resulted in a high metal dispersion and a good reactant flux in their tubular structure [50,51]. Therefore, the PANI/CNT is recommended to be used as the catalyst support instead of the commercial carbon for the use in a PEM fuel cell. According to all above results, although the PtCr/PANI-CNT catalyst exhibited a lower (4.98%) performance in the PEM fuel cell than PtPd/PANI-CNT, it exhibited a much lower (14.8%) ESA loss after 1000 cycles of repetitive CV. In addition, Cr is cheaper than Pd, and so PtCr/PANI-CNT is the recommended cathode catalyst for a PEM fuel cell.

## 4. Conclusions

A series of PtM (M = Ni, Co, Cr, Pd) catalysts on a PANI-CNT support were synthesized via an impregnation-seeding method. The Pt/PANI-CNT catalyst exhibited a higher ORR activity and stability than the commercial Pt/C catalyst although it had larger crystallite/particle sizes, lower catalyst dispersion and ESA. The addition of M enhanced the ORR activity and stability of the Pt/PANI-CNT catalyst, because the added M induced the formation of a PtM alloy and shifted the *d*-band center downfield, leading to a weak chemical interaction between oxygenated species and catalyst. Among the prepared PtM/PANI-CNT catalysts, the PtCr/PANI-CNT catalyst was optimal. Although it exhibited a slightly lower (~4.98%) performance in low temperature/pressure PEM fuel cell than the PtPd/PANI-CNT catalyst, it exhibited a much lower (~14.8%) ESA loss after 1000 cycles of repetitive CV. Thus, the PtCr/PANI-CNT is the recommended cathode catalyst for a PEM fuel cell.

## Figures and Tables

**Table 1:** Properties of the Pt/C and prepared PtM catalysts.

Catalyst	Lattice Parameter (nm)	Crystallite Size (nm)	Particle Size (nm)	Dispersion (*N_S_*/*N_T_*) (%) ^a^	Pt:M (Atomic Ratio) *^b^*	Metal Loading (wt %) *^b^*	Electrode Conductivity (S/cm)	ESA (m^2^/gPt)
Pt/C	0.3924	3.43	3.69 ± 0.56	32.76	100.00:0	17.4	18.21 ± 0.001	72.44
Pt/PANI-CNT	0.3923	6.29	4.75 ± 0.85	26.14	100.00:0	16.4	22.80 ± 0.001	69.77
PtNi/PANI-CNT	0.3909	6.76	5.80 ± 1.36	21.73	72.84:27.16	15.2	22.54 ± 0.001	34.44
PtCo/PANI-CNT	0.3899	6.58	6.08 ± 1.29	20.86	74.63:25.37	16.6	22.61 ± 0.001	44.87
PtCr/PANI-CNT	0.3909	6.72	5.82 ± 1.15	21.71	72.56:27.44	17.8	22.01 ± 0.003	66.88
PtPd/PANI-CNT	0.3901	6.33	6.06 ± 1.16	20.88	71.32:28.68	17.5	22.57 ± 0.002	68.36

*^a^* Calculated from the particle sizes derived from the TEM analysis; *^b^* Estimated from the EDX analysis.

**Table 2:** Physico-chemical properties of all prepared catalysts.

Catalyst	Pt Species	B.E. (eV)	Relative Peak Area (%)
Pt/C	Pt^0^	71.2	46.94
	Pt^2+^	72.4	38.53
	Pt^4+^	75.0	14.53
Pt/PANI-CNT	Pt^0^	71.3	51.14
	Pt^2+^	72.5	38.76
	Pt^4+^	74.9	10.10
PtNi/PANI-CNT	Pt^0^	72.0	56.82
	Pt^2+^	72.4	31.69
	Pt^4+^	74.9	11.49
	Ni^0^	852.6	48.37
	NiO	853.9	35.99
	Ni(OH)_2_	855.5	15.64
PtCo/PANI-CNT	Pt^0^	71.0	57.54
	Pt^2+^	72.4	31.48
	Pt^4+^	74.9	11.98
	Co	778.2	54.67
	CoO	783.5	11.98
	Co_3_O_4_	780	33.35
PtCr/PANI-CNT	Pt^0^	72.2	57.81
	Pt^2+^	72.7	31.75
	Pt^4+^	74.9	10.44
	Cr	574.3	59.37
	Cr(OH)_3_	575.5	28.49
	Cr_2_O_3_	579.5	12.15
PtPd/PANI-CNT	Pt^0^	71.3	60.12
	Pt^2+^	72.5	30.80
	Pt^4+^	75.0	9.08
	Pd	335.5	74.86
	PdO	336.7	25.14

**Table 3:** ORR activity of the different catalysts in a PEM fuel cell under a H_2_/O_2_ environment at 60 °C and ambient pressure.

Catalyst	Parameters Obtained from the NLLS Model							
	*j*_0.9 V_ (mA/cm^2^)	*j*_0.6 V_ (mA/cm^2^)	*−b* (mV/dec)	*j*_0_ (mA/cm^2^)	*R* (Ω cm^2^)	*n* (cm^2^/mA)	*m* (mV)	*R* ^2^
Pt/C	3.2	334	57.7	2.30 × 10^−5^	0.5400	0.0036	2.80	0.9998
Pt/PANI-CNT	3.6	350	57.0	2.47 × 10^−5^	0.5225	0.0034	3.55	0.9995
PtNi/PANI-CNT	4.4	364	57.0	2.68 × 10^−5^	0.5200	0.0032	3.40	0.9999
PtCo/PANI-CNT	4.8	378	56.0	2.65 × 10^−5^	0.5190	0.0031	3.24	0.9996
PtCr/PANI-CNT	5.2	382	57.5	4.38 × 10^−5^	0.5175	0.0029	3.20	0.9997
PtPd/PANI-CNT	5.4	402	57.5	5.14 × 10^−5^	0.5150	0.0028	3.60	0.9995

## Nomenclatures

[Pt]	Pt loading
*a*	lattice parameter
*b*	Tafel slope
*C*	bulk concentration of oxygen molecules
*D*	diffusion coefficient of oxygen
*E*_r_	reversible potential for the electrode
*F*	Faraday’s constant
*I*	test current
*j*	current density
*j*_0_	exchange current density for the ORR
*j*_k_	kinetic current density
*l*	number of layers and
*m*	parameters related to the mass transport limitation
*n*	parameters related to the mass overpotential
*n_e_*	number of exchanged electrons in the reaction
*N*_S_	number of surface atoms
*N*_T_	total number of atoms
*Q*_H_	charge exchanged
*q*_H_	charge required for the monolayer adsorption of H_2_ on Pt surfaces
*R*	total resistance
*s*	probe spacing
*t*	specimen thickness
*V*	measured voltage

## Symbols

ω	angular velocity
*ν*	kinematic viscosity
σ	in-plane conductivity
